# Alternating Hemiplegia of Childhood: Genotype–Phenotype Correlations in a Cohort of 39 Italian Patients

**DOI:** 10.3389/fneur.2021.658451

**Published:** 2021-04-08

**Authors:** Ramona Cordani, Michela Stagnaro, Livia Pisciotta, Francesco Danilo Tiziano, Maria Grazia Calevo, Lino Nobili, I.B.AHC Consortium, Elisa De Grandis

**Affiliations:** ^1^Department of Neurosciences, Rehabilitation, Ophthalmology, Genetics, Maternal and Child Health, University of Genoa, Genoa, Italy; ^2^Child Neuropsychiatry Unit, Department of Clinical and Surgical Neurosciences and Rehabilitation, Istituto di Ricovero e Cura a Carattere Scientifico Giannina Gaslini, Genova, Italy; ^3^Child Neuropsychiatry Unit, Azienda Socio Sanitaria Territoriale Fatebenefratelli- Sacco, Milano, Italy; ^4^Section of Genomic Medicine, Department of Life Science and Public Health, Catholic University of Sacred Heart, Roma, Italy; ^5^Epidemiology, Biostatistics and Committees Unit, Istituto di Ricovero e Cura a Carattere Scientifico Istituto Giannina Gaslini, Genoa, Italy

**Keywords:** alternating hemiplegia of childhood, ATP1A3, movement disorder, genotype, phenotype, flunarizine, epilepsy

## Abstract

Alternating hemiplegia of childhood is a rare neurological disease characterized by paroxysmal movement disorders and chronic neurological disturbances, with onset before 18 months of age. Mutations in the *ATP1A3* gene have been identified in up to 80% of patients. Thirty-nine patients [20 females, 19 males, mean age 25.32 years (7.52–49.34)] have been recruited through the Italian Biobank and Clinical Registry for Alternating Hemiplegia of Childhood. Demographic data, genotype, paroxysmal movement disorders, chronic neurological features, and response to flunarizine have been analyzed. *ATP1A3* gene mutations have been detected in 92.3% of patients. Patients have been divided into three groups—p.Asp801Asn mutation patients (26%), p.Glu815Lys cases (23%), and patients with other *ATP1A3* mutations—and statistically compared. The Italian cohort has a higher percentage of *ATP1A3* gene mutation than reported in literature (92.3%). Our data confirm a more severe phenotype in patients with p.Glu815Lys mutation, with an earlier age of onset of plegic (*p* = 0.02 in the correlation with other mutations) and tonic attacks. P.Glu815Lys patients most frequently present altered muscle tone, inability to walk (*p* = 0.01 comparing p.Glu815Lys and p.Asp801Asn mutations), epilepsy, and a more severe grade of dystonia (*p* < 0.05 comparing p.Glu815Lys and p.Asp801Asn mutations). They have moderate/severe intellectual disability and severe language impairment (*p* < 0.05). Interestingly, flunarizine seems to be more efficacious in patients with p.Glu815Lys mutation than p.Asp801Asn. In conclusion, our research suggests a genotype–phenotype correlation and provides information on this disorder's features, clinical course, and treatment.

## Introduction

Alternating Hemiplegia of Childhood (AHC) (OMIM #614820) is a rare and peculiar neurologic disorder, first described in 1971 (1). The annual incidence is <1/100,000 newborns (2). In AHC, a complex phenotype combines paroxysmal non-epileptic episodes, often triggered by contact with water, changes in temperature, physical or psychological stress, and epileptic seizures, both focal and generalized, with a high rate of refractory/super refractory status epilepticus (3). Developmental issues, cognitive deficits, neuropsychological impairments, and persistent neurologic disorders (2, 4–6) are expected hallmarks. In 1993, specific diagnostic criteria for AHC were first introduced (7) and then periodically updated. Aicardi's criteria for AHC (8) are (1) onset of paroxysmal events before 18 months of age; (2) repeated bouts of hemiplegia involving the right and left sides of the body during some attacks; (3) episodes of bilateral hemiplegia or quadriplegia starting either as a generalization of a hemiplegic episode or as bilateral from the start; (4) other paroxysmal disturbances including tonic/dystonic attacks, nystagmus, strabismus, dyspnea, and other autonomic phenomena occurring during hemiplegic bouts or in isolation; (5) immediate disappearance of all symptoms upon sleep, with probable recurrence of long-lasting bouts, 10–20 min after awakening; (6) evidence of developmental delay, intellectual disability, neurological abnormalities, choreoathetosis, and dystonia or ataxia; and (7) not attributable to other disorders. A great effort was made to understand the disease's genetic basis. In 2012, In 2012, two independent research groups - a group of German researchers (9) and an international consortium (10)—identified *de novo* heterozygous mutations in the *ATP1A3* gene performing next-generation sequencing to examine the genome of AHC patients (11). Next, this finding was replicated by an independent Japanese study that found *ATP1A3* mutations in patients with AHC (12). Currently, pathogenic variants in the *ATP1A3* gene are recognized as causing AHC in nearly 80% of patients (13). *ATP1A3* gene encodes for the alpha3 subunit of Na+/K+ATPase, which is neuron-specific. Interestingly, the cerebellum seems to be highly susceptive to *ATP1A3* dysfunction (14). Despite the growing number of pathogenic variants described, the most extensive cohort studies conducted in various populations showed that three variants account for ~60% of all cases. The p.Asp801Asn variant is detected in 30–43% of all cases, p.Glu815Lys is responsible for 16–35% of cases, and p.Gly947Arg is responsible for 8–15% (13, 15, 16). Mutations in the *ATP1A3* gene are associated with other neurological diseases such as rapid-onset dystonia-parkinsonism (DYT12, OMIM 128235) (9), cerebellar ataxia, areflexia, pes cavus, optic atrophy, sensorineural hearing loss (CAPOS) syndrome (OMIM # 601338) (17), the early infantile epilepsy with encephalopathy (EIEE) (18), and the recurrent encephalopathy with cerebellar ataxia (RECA) phenotype (19). Recently, mutations of the RHOBTB2 gene have been identified as responsible for a complex movement disorder with paroxysmal elements similar to AHC, suggesting to evaluate this gene in the diagnostic workup of patients with AHC, in particular those negative for ATPA1A3 mutations (20). Although a second major causative gene has not yet been identified, other gene mutations (*ATP1A2, CACNA1A, SLC1A3, SLC2A1*) have occasionally been related to AHC (21–24). Concerning pharmacological approaches, the main therapy goals are prophylaxis of paroxysmal attacks and chronic disturbance treatment, as dystonia and epilepsy. Disease-modifying therapy does not exist, albeit several drugs are administered as prophylaxis for paroxysmal attacks. The most frequent drugs used in AHC are flunarizine, benzodiazepines, carbamazepine, barbiturates, and valproic acid (25). It has been widely demonstrated that flunarizine induces a significant improvement in dystonic and plegic episodes (26).

In recent years, scientific efforts are focusing on defining a genotype–phenotype correlation to better understand the pathogenesis of the disease and provide indications for management and treatment. In literature, high interindividual variability as regards the clinical evolution has been reported. However, the investigation of adult patients' outcome excludes a progressive and neurodegenerative disorder (27). This study aims to describe an Italian cohort of AHC patients' genetic and clinical features and further establishes genotype–phenotype correlation. A significant number of patients enrolled for this study had already been included in previous multicenter studies published by Heinzen and colleagues in 2012 (10) and Panagiotakaki et al. in 2015 (13). However, in this research, we examined the clinical phenotype more in detail and flunarizine efficacy in different genotypes.

## Methods

### Sample Patients

Thirty-nine patients have been recruited through the I.B.AHC Italian Biobank and Clinical Registry for AHC (I.B.AHC). The diagnosis was based on Aicardi's criteria. All the patients have been diagnosed by consensus of the Scientific Association Committee of the Italian Association of Alternating Hemiplegia of Childhood (AISEA). Patients' referring pediatric neurologists have filled patients' forms, and I.B.AHC data managers validated the information's accuracy.

### Mutation Analysis

DNA was obtained from the blood of the probands and parents using standard procedures. *ATP1A3* mutations were identified by Sanger (34/39 patients), whole-exome (3/39), or next-generation sequencing for a targeted panel (2/39) for *ATP1A3* and other AHC-related genes. The mutation analysis was extended, whenever possible, to the parents to define if the mutation occurred *de novo*. Variant nomenclature was attributed based on RefSeq (NG_008015.1 and NM_152296, for genomic and mRNA sequences, respectively). Pathogenicity was revised following the original description, according to the ACMG criteria (28).

### Data Collection

Clinical, demographic, genetic, and pharmacological data of the 39 Italian patients have been collected. In more detail, data concerning paroxysmal movement disorders, particularly age of onset, type of episode as hemiplegic and tonic attacks, ocular abnormalities and autonomic dysfunctions, and the effect of sleep on episodes were examined. Chronic neurological features at last follow-up, especially muscle tone, gait characteristics, movement disorders (dystonia, chorea, myoclonus, postural or action tremor, bradykinesia), migraine, and the presence of intellectual disability and of varying degrees of language impairment, were also collected. The occurrence of reduced tone or hypertonia due to spasticity was defined “altered muscle tone.” For each patient, gait was assessed, and a differentiation was made between cases with normal gait, wide-based gait, and walking with trunk dystonia (“gait possible”), and subjects able to walk (“gait not possible”). Three degrees of dystonia severity have been identified by differentiating in mild dystonia when not interfering with function, moderate dystonia when interfering with activities in daily life, and severe dystonia when disabling. Intellectual disability was classified as “mild” (IQ of 50–69), “moderate” (IQ of 35–49), or “severe” (IQ <35). Language impairment was stated as mild when not affecting communication abilities, moderate when partially affecting communication abilities, or severe when affecting communication abilities. A distinction was made between AHC typical form (according to Aicardi's criteria for AHC) and atypical forms (24). This last definition was used in case of delayed occurrence of hemiplegic attacks, normal development, predominant dystonic phenotype, absence of quadriplegic attacks, or alternating upper-limb monoplegia. Lastly, the response to treatment with flunarizine was analyzed, considering as an improvement a reduction of at least 30% in duration, frequency, and intensity of the episodes.

### Statistical Analysis

Data are described as mean and standard deviation or median and range for continuous variables and as absolute and relative frequencies for categorical variables. Nonparametric analysis (Mann–Whitney *U*-test) for continuous variables and the Chi square or Fisher's exact test for categorical variables were used to measure differences between groups. Statistical significance was set as 0.017 using Bonferroni correction; all *p*-values were based on two-tailed tests. Statistical analysis was performed using *SPSS for Windows* (SPSS Inc., Chicago, Illinois USA). By meaning of *ATP1A3* mutation, patients were divided into three groups: patients with p.Asp801Asn mutation, patients with p.Glu815Lys mutation, and patients with other mutations, and statistically compared.

## Results

### Genetic Findings

*ATP1A3* gene mutations have been detected in 92.3% of patients (36/39): 23% (9/39) of patients had a p.Glu815Lys mutation, 26% (10/39) p.Asp801Asn mutation, and 43.6% (17/39) other various mutations. The more frequent gene mutation in the latter group was p.Gly947Arg (4/17; 10% of total patients). After revision of pathogenicity according to the ACMG guidelines (28), all variants were interpreted as Pathogenic (Class 5) since responding to the following criteria: PS1, PS3, PM2, PM6, PP2, PP3, and PP4.

Three out of 39 patients had no *ATP1A3* mutations. Tables 1, 2 summarize demographic data and paroxysmal and non-paroxysmal features in different group mutations.

**Table 1:** Demographic data and paroxysmal episode features in the three mutational groups.

	**Group 1:** **Various mutations** ***N* = 17**	**Group 2:** **p.Asp801Asn mutation** ***N* = 10**	**Group 3:** **p.Glu815Lys mutation** ***N* = 9**	***p* = 1 vs. 2**	***p* = 1 vs. 3**	***p* = 2 vs. 3**
**Age, years**						
Mean ± SD	20.6 ± 10.2	32.7 ± 10.6	25.1 ± 10			
Median (range)	18.5 (7.5–49.3)	32.2 (11.1–47.5)	22.4 (12.9–39.5)	**0.007**	0.26	0.13
	***N*** **(%)**	***N*** **(%)**	***N*** **(%)**			
Gender (M)	12 (70.6)	3 (30)	3 (33.3)	0.06	0.10	1
Typical AHC	13 (76.5)	10 (100)	5 (55.6)	0.26	0.38	0.03
PA onset, months	13.4 ± 9.1	6.8 ± 2.7	5.88 ± 0.83	0.07	0.02	0.26
TA onset, months	7.8 ± 8.1	11.1 ± 25.8	3.2 ± 2.3	0.40	0.12	0.84
EA onset, months	14.2 ± 38.2	2.4 ± 2.8	2.6 ± 2.5	0.42	0.37	0.96
Hemilateral PA	16 (94.1)	10 (100)	9 (100)	1	1	–
Total PA	11 (64.7)	6 (60)	6 (66.7)	1	1	1
Mixed PA	7 (43.8)	7 (70)	6 (66.7)	0.24	0.41	1
Tonic attacks	12 (75)	10 (100)	8 (88.9)	0.14	0.62	0.47
Hemilateral TA	7 (58.3)	7 (70)	4 (44.4)	0.67	0.67	0.37
Total TA	9 (69.2)	7 (70)	6 (66.7)	1	1	1
Autonomic dysfunction	11 (64.7)	9 (90)	7 (77.8)	0.20	0.67	0.58
Paroxysmal eye abnormalities	15 (88.2)	8 (80)	9 (100)	0.61	0.53	0.47
Sleep effect	16 (94.1)	8 (80)	7 (77.8)	0.53	0.27	1

*SD, standard deviation; AHC, alternating hemiplegia of childhood; PA, plegic attack; TA, tonic attack; EA, paroxysmal eye abnormalities. Bold values indicate statistically significant p values*.

**Table 2:** Epilepsy, non-paroxysmal movement disorders, and cognition in the three mutational groups.

	**Group 1:** **various mutations** ***N* = 17**	**Group 2:** **p.Asp801Asn mutation** ***N* = 10**	**Group 3:** **p.Glu815Lys mutation** ***N* = 9**	***p* = 1 vs. 2**	***p* = 1 vs. 3**	***p* = 2 vs. 3**
	***N*** **(%)**	***N*** **(%)**	***N*** **(%)**			
**Epilepsy**	9 (52.9)	6 (60)	8 (88.9)	1	0.09	0.30
**Status epilepticus**	2 (11.8)	2 (20)	4 (50)	0.61	0.06	0.32
**Muscle tone**						
Normal	3 (17.6)	3 (30)	–	0.64	0.53	0.21
Reduced/increased	14 (82.4)	7 (70)	9 (100)			
**Ataxia**	–	–	1 (11.1)		0.35	0.47
**Gait**						
Possible (normal/wide-based/trunk dystonia)	14 (82.4)	10 (100)	4 (44.4)	0.27	0.08	**0.01**
Not possible	3 (17.6)	–	5 (55.6)			
**Dystonia**	14 (82.4)	9 (90)	7 (77.8)	1	1	0.58
**Dystonia severity**						
No/mild	11 (64.7)	9 (100)	2 (25)	0.06	0.09	**0.002**
Moderate/severe	6 (35.3)	–	6 (75)			
**Myoclonus**	6 (35.3)	3 (30)	2 (22.2)	1	0.67	1
**Action/postural tremor**	2 (11.8)	1 (10)	3 (33.3)	1	0.30	0.30
**Chorea**	3 (17.6)	1 (10)	1 (13.9)	1	1	1
**Bradykinesia**	–	2 (20)	–	0.14	–	0.47
**Migraine**	8 (47.1)	5 (55.6)	3 (33.3)	1	0.68	0.64
**Intellectual disability**						
No/mild	8 (47.1)	4 (40)	–	1	0.02	0.09
Moderate/severe	9 (52.9)	5 (50)	9 (100)			
**Speech impairment**						
No/mild	9 (52.9)	7 (70)	–	0.45	**0.009**	**0.003**
Moderate/severe	8 (47.1)	3 (30)	9 (100)			

*Bold values indicate statistically significant p values*.

### Demographic Data

The average age of the whole group at the time of data collection was 18 ± 10.68 years (Table 1). In particular, patients with p.Glu815Lys mutation had 25.1 ± 10 years at the time of data collection, patients with p.Asp801Asn mutation 32.74 ± 10.6 years, and those with other mutations 20.6 ± 10.2 years. Comparing the different groups, the recruited patients with p.Asp801Asn mutation were significantly older than those with various mutations (*p* = 0.007). No differences concerning patient gender have been observed (20 female and 19 males). However, analyzing specific groups, a female prevalence in patients with p.Glu815Lys (67%) and p.Asp801Asn mutation (70%) has been detected. Vice versa, a male prevalence has been observed in the group with various mutations (71%), albeit without statistically significant differences (*p* = 0.06 comparing with p.Asp801Asn and *p* = 0.10 with p.Glu815Lys patients). All patients with p.Asp801Asn mutation had a typical AHC. Atypical AHC forms were more frequent in p.Glu815Lys (44 vs. 0% in cases with p.Asp801Asn- *p* = 0.03) and 23.5% in cases with other mutations (*p* = 0.26).

### Paroxysmal Features

Concerning paroxysmal events (Table 1), the onset of plegic episodes occurred at 5.88 ± 0.83 months in patients with p.Glu815Lys mutation, 6.8 ± 2.7 months in p.Asp801Asn patients, and 13.4 ± 9.1 months in patients with other mutations. The age of onset of plegic episodes was lower in p.Glu815Lys-mutated cases compared to the group with various mutations (*p* = 0.02). Tonic attacks appeared at 3.2 ± 2.3 months in p.Glu815Lys patients, at 11.1 ± 25.8 months in patients with p.Asp801Asn mutation, and 7.8 ± 8.1 months in patients with other mutations. Abnormal eye movements began at 2.6 ± 2.5 months in patients with p.Glu815Lys mutation, 2.4 ± 2.8 months in p.Asp801Asn patients, and 14.2 ± 38.2 months in patients with other mutations. No statistically significant differences were detected between the three groups as far as age of onset of paroxysmal attacks, albeit patients with p.Glu815Lys mutation globally presented an earlier onset than the other groups with a *p*-value approaching statistical significance. All patients with p.Glu815Lys and p.Asp801Asn mutations and 94% (16/17) with other mutations had hemilateral plegic attacks at follow-up. Moreover, from 60 to 67% of the mutated patients had total plegic attacks. All patients with p.Asp801Asn mutation, 89% (8/9) with p.Glu815Lys, and 75% (12/16) with other mutations had tonic attacks. Autonomic dysfunctions have been observed in 90% (9/10) of patients with p.Asp801Asn mutation, in 77.8% (7/9) of p.Glu815Lys patients, and in 64.7% (11/17) of other mutations. Lastly, abnormal eye movements have been detected in all p.Glu815Lys patients, in 80% (8/10) of patients with the p.Asp801Asn mutation, and 88% (15/17) of patients with other mutations. Symptoms were relieved by sleep in 78 to 94% of cases (the higher percentage in patients with other mutations, the lower in p.Glu815Lys mutation). Paroxysmal plegic and tonic attacks, autonomic dysfunctions, and paroxysmal eye movements have been widespread throughout the analyzed sample without statistically significant differences in the different groups.

### Non-paroxysmal Movement Disorders

Muscle tone was assessed by neurological examination. Statistical analysis compared patients with normal muscle tone to subjects with altered muscle tone (including both decreased and increased tone) in the three groups without finding any statistically significant difference (Table 2). Globally, reduced or increased muscle tone has been detected in 85% of the whole cohort (33/39, including 25 patients with hypotonia and eight with spastic hypertonia). Analyzing specific groups, all patients with p.Glu815Lys mutation (6/9 presented hypotonia and 3/9 hypertonia), 70% (7/10, 6 with hypotonia and 1 with hypertonia) with p.Asp801Asn, and 82.4% (14/17, 11 with reduced muscle tone and three with hypertonia) with other mutations presented abnormal muscle tone. At the last follow-up, 77% of patients walked independently. Twenty-eight percentage of them had wide-based gait, and 10% had trunk dystonia. Walking was not possible in 56% (5/9) of cases with p.Glu815Lys mutation and 17.6% (3/17) of patients with other mutations. Vice versa, gait was possible in all patients with the p.Asp801Asn mutation, although in some cases with dystonia and wide-based gait. The comparison of this latter group with the p.Glu815Lys mutated patients showed statistically significant values (*p* = 0.01). AHC Italian patients present a wide range of movement disorders, although they were not significantly different between the three groups (Table 2). Dystonia was observed in 85% of patients, most of whom had generalized dystonia (80% of cases). Patients with the highest percentage of dystonia were those with p.Asp801Asn mutation (90%, 9/10); lower percentages were found in p.Glu815Lys patients (78%, 7/9) and patients with other mutations (82%, 14/17). Statistical analysis compared cases with absent or mild dystonia with subjects with moderate or severe dystonia. Seventy-five percentage (6/8) of patients with the p.Glu815Lys mutation had moderate/severe dystonia, whereas all patients with p.Asp801Asn had a mild form; the difference in dystonia severity between p.Glu815Lys and p.Asp801Asn cases approached statistical significance (*p* = 0.002). On the other hand, dystonia in patients with various mutations was absent or mild in 64.7% (11/17) and moderate/severe in 35.3% (6/17) of cases. Myoclonus was noticed in 31% of patients ranging from 22% in p.Glu815Lys mutation to 35% in other mutations. Action and postural tremor were observed in 15.4% of cases: the higher percentage in p.Glu815Lys mutation (33%) and the lower percentage in p.Asp801Asn (10%).Chorea has been detected in 13% of mutated patients (17.6% of patients with various mutations, 10% of p.Asp801Asn cases, and 14% of patients with p.Glu815Lys mutation). Finally, bradykinesia has been detected only in the group of patients with p.Asp801Asn mutation (20, 5.4% of all patients).

### Epilepsy

As far as epilepsy is concerned (Table 2), 62% (24/39) of patients experienced epileptic seizures, both focal (37%) and generalized (26%). By meaning *ATP1A3* mutation, epilepsy was more frequent in cases with p.Glu815Lys mutation (89%, 8/9), in respect to 60% (6/10) with p.Asp801Asn and about 53% (9/17) with other mutations (*p* = 0.09 compared to the p.Glu815Lys mutation group). Besides, half of the patients with p.Glu815Lys mutation had a status epilepticus.

### Migraine

Forty-four percentage of all patients suffered from headache, with a higher percentage in the p.Asp801Asn mutation group (56%, 5/9—information not available for one patient), followed by the group with various mutations (47%, 8/17) (Table 2).

### Language and Cognitive Features

Regarding cognitive features (Table 2), a statistical comparison was made between the group with mild impairment and the group with moderate or severe intellectual disability. Moderate to severe impairment was observed in all patients with the p.Glu815Lys mutation. Lower percentages were found in other patients: 53% (9/17) of patients with other mutations (*p* = 0.02) and half of those with the p.Asp801Asn mutation (5/10) (*p* = 0.09). Furthermore, all p.Glu815Lys patients, 47% (8/17) with other mutations (*p* = 0.009) and 30% (3/10) of p.Asp801Asn patients (*p* = 0.003), exhibited moderate/severe speech impairment (absent or significantly reduced expressive language).

### Flunarizine Treatment Effect

Table 3 summarizes flunarizine effects in the three different mutational groups. Thirty-four patients received flunarizine (thirty-one patients with *ATP1A3* gene mutation and three patients without mutation). In patients with p.Glu815Lys mutation, this drug reduced the intensity and duration of episodes (>30%) in 67% (6/9) of cases and produced a decrease of frequency in 89% (8/9). Thirty-three of patients with p.Asp801Asn mutation (3/9) presented reduced intensity and frequency; 44% shorter duration. The 77% (10/13) of patients with other mutations had a reduction in episode intensity and the 85% (11/13) reduction in duration and frequency. Compared with the other two groups, patients with p.Asp801Asn mutations seemed to present a minor response to flunarizine; in particular, the episode frequency reduction was considerably lower than in other groups. In none of the patients examined, flunarizine produced a worsening of the clinical features.

**Table 3:** Flunarizine effect in the three mutational groups.

**Flunarizine effect**	**Group 1:** **Various mutations** ***N* = 13**	**Group 2:** **p.Asp801Asn mutation** ***N* = 9**	**Group 3:** **p.Glu815Lys mutation** ***N* = 9**	***p* = 1 vs. 2**	***p* = 1 vs. 3**	***p* = 2 vs. 3**
**Episode intensity**						
Reduction ≥30%	10 (76.9)	3 (33.3)	6 (66.7)	0.07	0.65	0.35
Reduction <30%/no effect	3 (23.1)	6 (66.7)	3 (33.3)			
**Episode duration**						
Reduction ≥30%	11 (84.6)	4 (44.4)	6 (66.7)	0.07	0.61	0.64
Reduction <30%/no effect	2 (15.4)	5 (55.6)	3 (33.3)			
**Episode frequency**						
Reduction ≥30%	11 (84.6)	3 (33.3)	8 (88.9)	0.03	1	0.05
Reduction <30%/no effect	2 (15.4)	6 (66.7)	1 (11.1)			

*The flunarizine effect on paroxysmal attacks was evaluated considering an improvement of at least 30% in the episodes' duration, frequency, and intensity*.

### Patients Without ATP1A3 Gene Mutation

The group of patients with no gene mutation exhibits somewhat heterogeneous clinical features. All patients had plegic attacks that developed from 0 to 24 months of age, abnormal paroxysmal eye movements, and total tonic attacks. In two out of three patients, paroxysmal events were relieved by sleep. One patient had epilepsy and status epilepticus, and all had dystonia, from mild to severe degree. In one patient, walking was not possible, and two patients showed a wide-based gait. They all had an intellectual disability (one mild and two moderate) and speech impairment (absent or significantly reduced expressive language). Finally, in one out of three patients, flunarizine resulted in a reduction of more than 30% in frequency, duration, and intensity of the episodes.

## Discussion

Our study reports genotype–phenotype correlation and provides information on paroxysmal and non-paroxysmal features, clinical course, and treatment of a cohort of 39 Italian AHC patients. The Italian patients have a higher percentage of *ATP1A3* gene mutation (92.3%) than as described in literature (13). The three most frequent mutations are p.Asp801Asn, p.Glu815Lys, and p.Gly947Arg, confirming findings already reported in the literature (15). Although an increasing number of pathogenic variants have been described, the variants listed above have been found to cause about 60% of all cases in the most extensive cohort studies. In particular, the p.Asp801Asn variant accounts for 30–43% of all cases, p.Glu815Lys 16–35% of cases, and p.Gly947Arg 8–15% (25). No other genes occasionally related to AHC patients (21–24) were detected in this case series. Previous studies showed a more severe phenotype in p.Glu815Lys mutation, with an earlier onset of symptoms, drug-resistant epilepsy, dystonia, and severe intellectual disability as main neurological findings (11, 13). Interestingly, a mouse model of AHC (*ATP1A3* ^E815K+/−^, Matoub, Matb ^+/−^) expressing the E815K mutation of the *ATP1A3* gene has recently been reported. In this study, the authors demonstrated that mutated mice exhibit features comparable to AHC patients with the same mutation. Mutated mice have poor motor initiative and profoundly impaired motor performance, particularly concerning coordination and abnormal gait. Of note, the hemiplegia and dystonia episodes were both spontaneous and induced by a high level of stress, similar to the human phenotype. Besides, the mouse model presented spontaneous seizures or induced seizures and sudden unexpected death in epilepsy (SUDEP). Although there are differences between the proposed models, some features are common: brain hyperexcitability, motor abnormalities (spontaneous or provoked), and behavioral alterations, according to a fundamental role of *ATP1A3* in brain functioning (29). In our sample, patients with p.Glu815Lys mutation most frequently present altered muscle tone, inability to walk (*p* = 0.01 comparing p.Glu815Lys and p.Asp801Asn mutations), epilepsy, and a more severe grade of dystonia (*p* = 0.002 comparing p.Glu815Lys and p.Asp801Asn mutations), confirming a worse phenotype related to this mutation. Moreover, these patients have moderate/severe intellectual disability and moderate/severe language impairment compared to other groups. In particular, the language appears more injured in this group, reaching significant values in statistical analysis (*p* = 0.009 and *p* = 0.003). Regarding paroxysmal episodes, our data show that p.Glu815Lys patients present an earlier age of onset of plegic (*p* = 0.02 in the correlation with other mutations) and tonic attacks. Furthermore, four out of nine of our p.Glu815Lys patients presented rapid motor decline and language loss and a sudden worsening of dystonia. As far as psychomotor deterioration is concerned, we assume that a genetic determinant could be involved considering that severe deterioration with no recovery is typical of Rapid Onset Dystonia Parkinsonism, another allelic disorder caused by the *ATP1A3* mutation. Panagiotakaki and colleagues (13) after reviewing clinical data of a large cohort of 155 patients from the International Consortium of AHC reported a milder phenotype in p.Asp801Asn patients with later onset of the paroxysmal events, moderate intellectual disability, and a higher behavioral problems rate. Similarly, in our study, patients with this mutation had milder clinical signs than the p.Glu815Lys group. All patients had independent walking, and a less percentage of cases had alterations in muscle tone on neurological examination. Besides, ataxia, chorea, and action and postural tremor were less frequent than in other groups. However, a higher prevalence of dystonia was observed than in other groups (90% of cases), although no patients had moderate/severe forms (common finding in patients with the p.Glu815Lys mutation, *p* = 0.002). As for the cognitive aspects, they had a lower percentage of moderate/severe intellectual disability. The group with various mutations exhibited a later onset of paroxysmal hemiplegic attacks and abnormal eye movements. Concerning non-paroxysmal neurologic features, a more severe neurological impairment than patients with p.Asp801Asn mutation has been detected. Interestingly, in this group, a male prevalence was observed differently from other groups. It is well known that AHC patients harboring *ATP1A3* mutations exhibit various forms of hyperkinetic non-paroxysmal movement disorders as chorea, dystonia, myoclonus, and ataxia, with a heterogeneous degree of severity. Our study does not confirm previous study results. In fact, in French patients, choreoathetosis and ataxia have been described in all cases (7), and in US patients, ataxia was reported in 68% of cases (8). In a recent research, a lower rate of non-paroxysmal movement disorders has been reported (30). It is reasonable to hypothesize that these differences between studies are partly due to the complexity in assessing movement disorders' phenomenology. Further, this is the first study aiming to analyze flunarizine's effect on paroxysmal episodes in different mutational groups. Flunarizine, a calcium antagonist, reduces the duration, severity, and frequency of paroxysmal attacks in up to 80% of cases in some studies (8, 26). The exact mechanism by which flunarizine improves paroxysmal spells in AHC is not yet fully recognized. Flunarizine may be useful in patients with Na+/K+ pump dysfunction by blocking voltage-gated Na+ and Ca+ currents, limiting neuronal hyperexcitability, and increasing the threshold to develop cortical spreading depression (29). Our study confirms the benefit of flunarizine on paroxysmal episodes and highlights differences of effects on different mutations. Comparing the two most frequent *ATP1A3* mutations, flunarizine seems to be more efficacious in patients with p.Glu815Lys mutation than p.Asp801Asn, in reducing intensity, frequency, and duration of paroxysmal episodes. Interestingly, Helseth et al. demonstrated that flunarizine also reduced hemiplegic attacks and seizures in E815K (p.Glu815Lys) mouse models (29). A positive response to flunarizine treatment was also observed in the group of patients with various mutations. Greater efficacy of reducing the intensity, duration, and frequency of paroxysmal episodes than in the p.Asp801Asn mutation group has been recorded, although no statistically significant differences have been achieved. Moreover, even if the difference did not reach a significant value, a greater efficacy on reducing intensity and duration of episodes was observed in this group than in p.Glu815Lys patients. These results are not easily interpretable, given the numerous variables involved in response to treatment. Future studies may further clarify the relationship between genotype and treatment response. Understanding the molecular pathogenesis and the neuronal circuits involved in the disease would guide the therapeutic choices. Alternating hemiplegia of childhood is a heterogeneous disorder characterized by dysfunctions involving different neuronal networks, causing paroxysmal and non-paroxysmal movement disorders, epilepsy, and cognitive deficits. Our results confirm that different *ATP1A3* gene mutations are related to different phenotypic features. Due to the disease's rarity, the sample size may have limited the study's statistical power. This limitation mainly concerns the group of patients with various mutations, including p.Gly947Arg mutation, for which it was not possible to conduct separate statistical analyzes. Further studies involving a more extensive patient sample could better clarify these differences and increase knowledge about gene functions and different mutations' role. Another possible bias is related to the wide age range of the patients' cohort and to the different mean ages in which the 3 groups were studied. However, we assume that these variations did not alter the clinical data comparison, considering that literature data seem to exclude a degenerative course in AHC and a clear progression of symptoms over the years (27).

Prediction of the disease's development and severity using genotype information is the primary aim of genotype–phenotype correlation studies. This knowledge is necessary to evaluate new therapeutic approaches, provide a more accurate prognosis to patients and caregivers, and develop the most appropriate care management guidelines. An increasingly in-depth knowledge of the disease in its clinical and genetic aspects will allow for more effective management and treatment.

## Data Availability Statement

Data of mutations reported within this study have been deposited in Leiden Open Variation Database (databases.lovd.nl/shared/references/DOI:10.3389/fneur.2021.658451).

## I.B.AHC Consortium

Maria Teresa Bassi, Claudio Zucca, Edvige Veneselli, Elisa De Grandis, Michela Stagnaro, Filippo Franchini, Maria Rosaria Vavassori, Melania Giannotta, Giuseppe Gobbi, Tiziana Granata, Nardo Nardocci, Francesca Ragona, Emanuela Abiusi, Agnese Novelli, Fiorella Gurrieri, Giovanni Neri, Francesco Danilo Tiziano, Federico Vigevano, Alessandro Capuano, Stefano Sartori.

## Author Contributions

RC, MS, and EDG: conception and design of the study. RC, MS, LP, MC, I.B.AHC Consortium, and EDG: acquisition and analysis. RC, MS, LP, MC, LN, and EDG: drafting of the manuscript or tables. All authors contributed to the article and approved the submitted version.

## Conflict of Interest

The authors declare that the research was conducted in the absence of any commercial or financial relationships that could be construed as a potential conflict of interest.
